# Compassion fatigue and compassion satisfaction among nurses/midwives caring parents with pregnancy loss or infertility: a cross-sectional study

**DOI:** 10.3389/fpubh.2025.1668647

**Published:** 2025-10-23

**Authors:** Siyu Liu, Hongmei Han, Rukmali Athurupana, Titi Yang, Mikiya Nakatsuka

**Affiliations:** ^1^School of Public Health and Nursing, Hangzhou Normal University, Hangzhou, China; ^2^Graduate School of Health Sciences, Okayama University, Okayama, Japan; ^3^Graduate School of Medicine, Dentistry and Pharmaceutical Sciences, Okayama University, Okayama, Japan; ^4^Reproduction Center, Okayama University Hospital, Okayama, Japan

**Keywords:** compassion fatigue, burnout, nurses, infertility, spontaneous abortion

## Abstract

**Objectives:**

The study aimed to assess the prevalence and potential factors associated with compassion fatigue and compassion satisfaction among nurses/midwives who support parents with pregnancy loss or infertility.

**Methods:**

A total of 370 nurses and midwives were recruited from 43 hospitals and clinics in the Chugoku-Shikoku region of Japan. We collected data using a demographic survey, the Professional Quality of Life Scale, and the Adolescent Resilience Scale. Descriptive statistics, *t*-tests, one-way ANOVA, Pearson correlation analyses, and multiple linear regression were conducted for data analysis.

**Results:**

The participants reported moderate level for compassion satisfaction (83.8%) and burnout (77.3%), and a low level for secondary traumatic stress (51.4%). The predictors explained 42.9% predicted 42.9, 49.1, and 16.9% of the variance in the model of compassion satisfaction, burnout, and secondary traumatic stress, respectively.

**Conclusion:**

Nurses/midwives demonstrated moderate levels of burnout and low levels of secondary traumatic stress. Resilience, marital status, education background, working characteristics, organizational support, knowledge sufficiency, and the practice of grief care were identified as factors associated with compassion fatigue and compassion satisfaction among nurses/midwives.

## Introduction

Compassion fatigue (CF), which encompasses burnout (BO) and secondary traumatic stress (STS), refers to emotional, physical, and psychological exhaustion caused by prolonged and intense work-related stress exposure of healthcare providers (1). In contrast, compassion satisfaction (CS) reflects the positive fulfillment derived from helping others (1). Together, CF and CS constitute the two dimensions of professional quality of life (ProQoL) (1).

Pregnancy loss—including miscarriage (loss before viability, occurring in 15.3% of pregnancies) and stillbirth (fetal loss after 22 weeks)—affects millions of families globally (2). In Japan, stillbirth is defined as the end of pregnancy after 22 weeks of gestation, with a reported rate of 2.7 per 1,000 births and the miscarriage rate before 22 weeks of pregnancy reached 8–15% in 2022 (3). Infertility is characterized by the inability to conceive after 12 months of regular unprotected intercourse and affects around one in seven couples (4). In Japan, approximately 18.2% of couples, or 1 in 5.5, have undergone or are currently undergoing infertility treatment or testing (5). Both conditions are associated with significant psychological distress, including depression, anxiety, and social stigma (6, 7).

Nurses providing care in these contexts are routinely exposed to profound grief and emotional distress (8, 9). Grief care (GC) aims to support parents who have experienced pregnancy loss by focusing on respectful and supportive measures, such as shared decision-making, effective communication, and acknowledgment of their parental role (8, 9). In the context of infertility, patient-centered care is highly valued and encompasses a comprehensive range of services. This approach involves addressing common inquiries, providing support during distressing events such as cycle disruptions and negative pregnancy tests, offering resources on stress management, delivering cost-effective treatments, and facilitating opportunities for engagement with support groups (10).

Although CF and CS have been extensively studied in high-stress specialties like ICU (11), emergency (12), oncology (13), and pediatrics (14), little attention has been given to nurses caring for pregnancy loss and infertility populations. Studies in gynecology and obstetrics (OB/GYN) clinics/hospitals indicate CF prevalence rates as high as 75.9%, influenced by factors including emotional labor, lack of professional efficacy, and organizational culture (15). Protective factors such as resilience, clinical supervision, and compassionate workplace support have been identified, with meta-analytic evidence confirming a significant negative correlation between resilience and burnout-related symptoms (16, 17).

Given the significant emotional burden associated with supporting individuals through pregnancy loss and infertility, along with the lack of targeted research in this nursing population, there is a critical need to investigate the prevalence, predictors, and outcomes of CF and CS in this context.

## Aims

The objective of this study was to examine the prevalence and potential factors associated with compassion fatigue and compassion satisfaction among nurses/midwives supporting parents with pregnancy loss or infertility.

### Design and participants

A cross-sectional study was conducted using a convenience sampling method. Nurses and midwives were recruited from 43 hospitals and clinics in the Chugoku-Shikoku region (including Tottori, Shimane, Okayama, Hiroshima, Yamaguchi, Tokushima, Kagawa, Ehime, and Kochi prefectures) of Japan between January and April 2022.

Inclusion criteria were: (1) being a registered nurse or midwife; (2) having at least 1 year of clinical experience; and (3) providing care to patients experiencing pregnancy loss (miscarriage or stillbirth) or infertility at the time of the study. There were no exclusion criteria based on age, gender, or specific workplace setting, as we aimed to capture a broad perspective of the target population.

### Sampling and data collection

All hospitals and clinics in the Chugoku-Shikoku region that admitted women who had experienced pregnancy loss or infertility were identified via their official websites. Settings include Assisted reproductive technology (ART) clinics provide infertility services and treatments such as *in vitro* fertilization (IVF). OB/GYN hospitals/clinics refer to medical facilities that specialize in providing healthcare services related to women’s reproductive health. General hospitals are medical facilities that provide a wide range of healthcare services (referred to herein as university-affiliated hospitals or general hospitals).

The heads of nursing departments or chief midwives at 202 institutions were initially contacted by mail to explain the study purpose and request participation. Forty-three institutions agreed to cooperate and distributed the survey to their nursing staff. A total of 503 survey packets were distributed. Prospective participants received the questionnaire package, which included a cover letter explaining the study’s purpose, a statement ensuring anonymity and voluntary participation, the survey forms, and a return envelope.

The cover letter served as the informed consent document. Participants were informed that returning the completed questionnaire implied their consent to participate. Data were anonymized to ensure confidentiality, and participants were assured that their responses would be used solely for research purposes.

A total of 388 questionnaires were returned (response rate: 77.1%). After excluding 18 questionnaires with more than 50% of items missing, 370 valid responses were included in the final analysis (valid response rate: 95.4% of returned surveys).

### Ethical considerations

The study was conducted in accordance with the Declaration of Helsinki and received ethical approval from the Medicine Ethics Committee of Okayama University Hospital (Approval No. 2201-292).

## Measures

### Demographic characteristics

The survey questions encompassed the respondent’s demographic characteristics (age, gender, marital status, and educational background), gestational history, average working hours per week in the past year, and number of patients with miscarriage and stillbirth in the past year with close and open-ended questions. Supporting from organizations was included. Participants were also asked to report whether they had experienced the following events: childbirth, miscarriage, stillbirth, death of children and other close persons and infertility treatment. The knowledge and learning experience of GC and practice of GC were also included.

### The ProQoL

The Professional Quality of Life Scale (ProQoL) consists of three subscales: CS, BO and STS (1). The instrument is a 30-item self-report instrument that uses a 5-point Likert scale (1 = Never, 5 = Very often) (1). Items 1, 4, 15, 17 and 29 were reverse scored. Each subscale score ranges from 0 to 50. Higher scores for each sub-scale indicate higher levels of CS, BO and STS. According to (1), cutoff rates are low = 22 or lower, average = 23–41 and high = 42 or higher. The ProQoL’s Cronbach’s *α* values of 0.87, 0.72 and 0.80 for CS, BO and STS, respectively. The Japanese version of the ProQoL scale was used in this study (18). This version is similar to the English version in terms of the item subscales and scoring method. In this study, the Cronbach’s alpha of the ProQoL scale was 0.66, 0.92 for the CS subscale, 0.68 for the BO subscale, and 0.81 for the STS subscales.

### Adolescent resilience scale

The Adolescent Resilience Scale (ARS) consists of 21 items and three factors: Novelty Seeking, Emotional Regulation, and Positive Future Orientation, using a 5-point Likert scale (5 = Definitely yes, 1 = Definitely no). The Cronbach’s alpha for the total score was 0.85, Novelty Seeking 0.79, Emotional Regulation 0.77, and Positive Future Orientation 0.81 (19). Although initially developed for adolescents, the core constructs of resilience measured by the ARS (Novelty Seeking, Emotional Regulation, Positive Future Orientation) are considered stable personality traits applicable across the lifespan and relevant to adaptive functioning in high-stress professions like nursing. The scale’s use has been successfully deployed in nursing populations, with established studies confirming its good validity and reliability for this demographic (20, 21). In this study, the Cronbach’s alpha for the total score was 0.91, Novelty Seeking 0.84, Emotional Regulation 0.81, and Positive Future Orientation 0.83.

## Data analysis

The study results were analyzed using SPSS (Statistical Package for Social Sciences) Statistics 27.0. Variables with more than 20% missing data were excluded from the analysis to prevent potential bias in the results. For variables with less than 20% missing data, we applied multiple imputation methods to handle the missing values, thereby ensuring data integrity and reliability. Descriptive statistics were used to describe the basic characteristics of participants and the prevalence of CS, BO, and STS. A cut-off score of knowledge, working years, working hours/week, number of childbirth, support from the organization were determined based on a median split of the sample. The distribution of the data was tested first by using descriptive analyses. Then, univariate analysis and Pearson’s correlations were conducted to examine the differences between the dependent and independent variables. For data with a normal distribution (BO), independent *t*-tests and one-way ANOVA were used. For data with a non-normal distribution (CS and STS), the Mann–Whitney *U* test and Kruskal–Wallis *H* test were employed. Significant variables (*p* < 0.05) identified in the univariate analysis and in Pearson’s correlations were then entered into the multiple linear regression to examine the unique contribution of each potential independent variable to the dependent variables (CS, BO, STS).

## Results

### Demographics of participants

The final analytical sample consisted of 370 valid surveys. These were derived from an initial pool of 388 returned surveys (from 503 distributed, a 77.1% return rate), after the exclusion of 18 surveys with more than 50% of items missing. This resulted in a valid response rate of 95.4% from the returned surveys. The majority of the participants were females (99.7%). The respondents’ median [range] age was 42 [23–65] years; 67.3% were married, 65.4% had graduated from nursing school, and 71.9% had a midwifery license (see Table 1).

**Table 1:** Characteristics of study population.

Characteristics	Category	Total (*n* = 370)	ART clinics (*n* = 57)^a^	OB/GYN hospitals/clinics (*n* = 118)^b^	General hospitals (*n* = 195)^c^	*p*-value	Multiple comparisons
Age		42 [23–65]	40.5 [26–65]	46 [27–65]	39 [23–65]	0.000†	b vs. c <0.001§
Gender	Woman	369 (99.7%)	56 (98.2%)	118 (100%)	195 (100%)		
Marital status	Single	92 (24.9%)	14 (24.6%)	12 (10.2%)	66 (33.8%)	0.000‡	a vs. b <0.05, b vs. c <0.001§
	Married	249 (67.3%)	36 (63.2%)	94 (79.7%)	119 (61%)		
	Divorced or widowed	29 (7.8%)	7 (12.3%)	12 (10.2%)	10 (5.1%)		
Fertility-related experience	Childbirth	239 (64.6%)	34 (59.6%)	92 (78.0%)	113 (57.9%)	0.001‡	a vs. b <0.05, b vs. c <0.001§
	Miscarriage	85 (23.0%)	6 (10.5%)	38 (32.2%)	41 (21.0%)	0.004‡	a vs. b <0.005, b vs. c <0.05§
	Stillbirth	6 (1.6%)	0 (0%)	4 (3.4%)	2 (1%)	0.165‡	
	Infertility	59 (15.9%)	9 (15.8%)	24 (20.3%)	26 (13.3%)	0.260‡	
	Recurrent pregnancy loss	26 (7%)	6 (10.5%)	11 (9.3%)	9 (4.6%)	0.153‡	
Death experience	Death of own child	2 (0.5%)	0 (0%)	1 (0.8%)	1 (0.5%)	0.691‡	
	Death of familiar person	88 (23.8%)	16 (28.1%)	28 (23.7%)	44 (22.6%)	0.716‡	
Education background	Nursing school	242 (65.4%)	47 (82.5%)	79 (66.9%)	116 (59.5%)	0.005‡	a vs. b <0.05, a vs. c <0.001§
	University and above	128 (34.6%)	10 (17.5%)	39 (33.5%)	79 (40.5%)		a vs. c <0.01§
Qualifications	Nurse	104 (28.1%)	44 (77.2%)	36 (30.5%)	24 (12.3%)	0.000‡	a vs. b, a vs. c, b vs. c <0.001§
	Midwife	266 (71.9%)	13 (22.8%)	82 (69.5%)	171 (87.7%)		
Work setting	Outpatient	69 (18.6%)	42 (73.7%)	17 (14.4%)	10 (5.1%)	0.000‡	a vs. b, a vs. c, b vs. c <0.001§
	Inpatient	119 (32.2%)	0 (0%)	19 (16.1%)	100 (51.3%)		a vs. b, a vs. c, b vs. c <0.01§
	Both	182 (49.2%)	15 (26.3%)	82 (69.5%)	85 (43.6%)		a vs. b, a vs. c, b vs. c <0.01§
Work years		7.9 [0.1–35]	4.5 [0.5–28.3]	7.5 [0.1–35.0]	9 [0.5–34.9]	0.011†	a vs. b <0.01, a vs. c <0.005§
Work hours/week		40 [10.0–61.5]	40 [16–53.9]	40 [12–56]	40 [24–61.5]	0.007†	a vs. c <0.01, b vs. c <0.05§
Teamwork	Have	219 (59.2%)	16 (28.1%)	65 (55.1%)	138 (70.8%)	0.000‡	a vs. b, a vs. c <0.001, b vs. c <0.005§
	Not have	151 (40.8%)	41 (70.9%)	53 (44.9%)	57 (29.2%)		
Support from organization		1 [0–3]	7 (12.3%)	12 (10.2%)	10 (5.1%)	0.120‡	
Knowledge of perinatal loss		5 [0–6]				0.001‡	a vs. c, b vs. c <0.01§
Knowledge of grief care		9 [0–11]	0 (0%)	4 (3.4%)	2 (1%)	0.385‡	
Learning experience (school)	Yes	158 (42.7%)	42 (73.7%)	47 (39.8%)	96 (49.2%)	0.007‡	a vs. c <0.01§
	No	212 (57.3%)	15 (26.3%)	71 (60.2%)	99 (50.8%)		
Learning experience (work)	Yes	243 (65.7%)	28 (49.1%)	76 (64.4%)	139 (71.3%)	0.005‡	a vs. b <0.001, a vs. c <0.005§
	No	127 (34.3%)	29 (50.9%)	42 (35.6%)	56 (28.7%)		

median [range], *N* (%), †: Kruskal–Wallis test, ‡: *χ*^2^-test/Fisher’s exact test, §: post-hoc test by Bonferroni; ^a^ART clinics; ^b^Gynecology and obstetrics clinics/hospitals; ^c^General hospitals.

### ProQol and resilience

The CS and BO average score of the survey was moderate, and STS was low (see Table 2). Most of the participants indicated moderate level for CS (83.8%) and BO (77.3%) and low level for STS (51.4%). There were no high-level of STS in this study. The resilience scores ranged from 38 to 104 with a mean score of 70.2 (Table 2).

**Table 2:** Degree of quality of life (ProQol) scores and frequencies and resilience scores (*n* = 370).

Variables	Score category	*N*	%	Total score
Compassion satisfaction	High (≥42)	35	9.5	32.8 ± 6.9
	Moderate (23–41)	310	83.8	33 [10–50]
	Low (<23)	25	6.8	
Burnout	High (≥42)	3	0.8	27.2 ± 5.7
	Moderate (23–41)	286	77.3	27 [12–45]
	Low (<23)	81	21.9	
Secondary traumatic stress	High (≥42)	0	0	22.4 ± 5.5
	Moderate (23–41)	180	48.6	22 [10–41]
	Low (<23)	190	51.4	
Resilience	–	–	–	70.2 ± 11.7
				70 [38–104]

mean ± S.D.

median [range].

### Correlations between ProQol and resilience

A higher resilience level was correlated with higher CS scores (*r* = 0.564, *p* < 0.001) but with lower BO scores (*r* = −0.615, *p* < 0.001) and STS (*r* = −0.375, *p* < 0.001). (Table 3)

**Table 3:** Correlation among compassion satisfaction, burnout, secondary traumatic stress, and resilience (*n* = 370).

	Compassion satisfaction	Burnout	Secondary traumatic stress	Resilience
Compassion satisfaction	1			
Burnout	−0.700*	1		
Secondary traumatic stress	−0.217*	0.558*	1	
Resilience	0.564*	−0.615*	−0.375*	1

*p < 0.05.

### Univariate analysis results

The following groups of respondents demonstrated significantly higher levels of CS (Table 4): those who were married (*p* ≤ 0.001), worked in OB/GYN hospitals/clinics (*p* ≤ 0.001), had ≥20 years of care provision experience (*p* = 0.008), had childbirth experience (*p* ≤ 0.001), had given birth ≥3 times (*p* = 0.006), had experienced miscarriage (*p* = 0.026), reported no traumatic experiences (*p* = 0.001), had undergone infertility treatment (*p* = 0.019), received ≥2 types of organizational support (*p* ≤ 0.001), had a perinatal loss knowledge score ≥5 (*p* = 0.019), had a GC knowledge score ≥9 (*p* = 0.001) and had workplace learning experience (*p* = 0.028). Additionally, higher CS was significantly associated with practices such as “providing opportunities to create memories with the baby” (*p* = 0.001), “being aware of physical and emotional exhaustion” (*p* = 0.044), “showing consideration for physical pain and empathy for sadness and grief” (*p* = 0.004), and “providing follow-up care after hospital discharge” (*p* = 0.007) (Table 4).

**Table 4:** Univariate analyses of the sociodemographic factors associated with compassion satisfaction, burnout, and secondary traumatic stress (*N* = 370).

Characteristics	Category	Compassion satisfaction			Burnout			Secondary traumatic stress		
		*M* (P25, P75)	*Z*/*F*	*P-*Value	Mean (SD)	*t*/*F*	*P-*Value	*M* (P25, P75)	*Z*/*F*	*P-*Value
Marital status	Single	26.00, 35.00	16.243	0.000	29.70 ± 5.274	12.968	0.000	19.25, 28.00	7.710	0.021
	Married	29.00, 39.00			26.37 ± 5.695			18.00, 25.00		
	Divorced or widowed	30.00, 37.50			25.83 ± 5.015			18.00, 25.00		
Educational background	Below university	29.00, 38.00	−1.193	0.233	27.16 ± 5.708	0.169	0.984	19.00, 26.00	−1.549	0.121
	University and above	27.00, 37.75			27.14 ± 5.766			18.00, 25.00		
Qualification	Midwives	29.00, 38.00	−0.358	0.720	27.23 ± 5.560	2.228	0.697	19.00, 26.00	−0.468	0.640
	Nurses	28.25, 38.00			26.97 ± 6.137			19.00, 25.00		
Facilities	ART clinic	26.00, 38.00	37.343	0.000	27.46 ± 6.217	31.715	0.000	18.50, 25.00	12.521	0.002
	OB/GYN hospitals/clinics	31.75, 40.00			24.04 ± 4.896			17.00, 25.00		
	General hospital	27.00, 36.00			29.04 ± 0.368			19.00, 27.00		
Work forms	Outpatient	27.00, 36.50	3.315	0.191	27.17 ± 6.331	1.390	0.138	19.00, 25.00	2.997	0.224
	Inpatient	28.00, 37.00			27.83 ± 5.043			19.00, 27.00		
	Both	30.00, 39.00			26.71 ± 5.880			18.00, 25.00		
Years as care provider	≤10	28.00, 37.00	9.552	0.008	27.35 ± 5.704	1.218	0.297	18.00, 26.00	0.410	0.815
	10–20	28.00, 38.00			27.38 ± 6.239			19.00, 25.00		
	>20	30.00, 39.50			26.07 ± 4.855			19.00, 27.50		
Work hours/week	≤40	29.00, 38.00	−0.195	0.846	27.03 ± 5.649	0.131	0.292	19.00, 26.00	−1.224	0.221
	>40	29.00, 37.50			27.92 ± 6.130			19.00, 24.00		
Personal experience										
Childbirth	Have	30.00, 39.00	−4.022	0.000	26.29 ± 5.776	1.511	0.000	18.00, 25.00	−2.279	0.023
	Not have	27.00, 35.00			28.73 ± 5.285			19.00, 27.00		
Number of childbirths	<3	28.00, 38.00	−2.742	0.006	27.60 ± 5.687	0.084	0.013	19.00, 26.00	−1.493	0.135
	≥3	30.00, 39.25			25.93 ± 5.663			18.75, 25.00		
Miscarriage	Have	30.00, 39.00	−2.228	0.026	26.08 ± 5.797	0.252	0.048	18.00, 26.00	−0.281	0.779
	Not have	28.00, 37.00			27.48 ± 5.668			19.00, 26.00		
Recurrent pregnancy loss	Have	29.00, 33.50	−1.748	0.080	27.35 ± 4.516	2.567	0.861	19.00, 26.00	−0.198	0.843
	Not have	29.00, 38.00			27.14 ± 5.806			19.00, 26.00		
Death of child	Have	31.00	−0.322	0.747	27.00 ± 0.000	3.570	0.969	19.00 -	−0.060	0.952
	Not have	29.00, 38.00			27.16 ± 5.736			19.00, 26.00		
Death of close persons	Have	28.00, 39.75	−0.133	0.894	27.04 ± 5.510	2.464	0.479	19.00, 26.00	−0.182	0.856
	Not have	29.00, 38.00			27.53 ± 6.368			19.00, 26.00		
None of them	Yes	29.00, 39.00	−3.194	0.001	28.56 ± 4.815	5.513	0.004	18.00, 26.00	−1.335	0.182
	No	27.00, 35.00			26.65 ± 5.944			19.00, 26.00		
Infertility treatment	Have	30.00, 39.00	−2.351	0.019	26.17 ± 5.902	0.292	0.148	18.00, 25.00	−1.250	0.211
	Not have	28.00, 37.00			27.34 ± 5.676			19.00, 26.00		
Teamwork	Have	28.00, 37.00	−0.246	0.806	27.14 ± 5.899	0.484	0.936	18.00, 25.00	−1.315	0.189
	Not have	29.00, 38.00			27.19 ± 5.470			19.00, 26.00		
Support from organization	<2	28.00, 37.00	−4.120	0.000	27.45 ± 5.807	5.226	0.004	18.00, 26.00	−0.648	0.517
	≥2	34.25, 40.00			24.73 ± 4.285			19.00, 24.00		
Knowledge of perinatal loss	<5	28.00, 37.00	−2.341	0.019	28.30 ± 6.104	1.534	0.005	20.00, 28.00	−1.061	0.289
	≥5	29.00, 39.00			26.56 ± 5.427			19.00, 26.00		
Knowledge of grief care	<9	28.00, 36.00	−3.402	0.001	27.66 ± 5.816	0.105	0.146	18.00, 26.00	−0.019	0.985
	≥9	30.00, 39.00			26.78 ± 5.633			19.00, 26.00		
Learning experience (school)	Yes	28.50, 38.00	−1.402	0.161	27.70 ± 5.534	0.682	0.113	19.00, 26.00	−1.334	0.182
	No	29.00, 39.00			26.75 ± 5.834			18.00, 25.00		
Learning experience (work)	Yes	29.00, 39.00	−2.198	0.028	26.74 ± 5.632	0.314	0.055	19.00, 26.00	−0.376	0.707
	No	27.00, 37.00			27.94 ± 5.827			19.00, 25.00		
Provide opportunities to make memories with the baby	Yes	29.00, 39.00	−3.246	0.001	26.91 ± 5.730	0.036	0.006	18.25, 25.00	−0.340	0.734
	No	26.00, 34.75			28.45 ± 5.540			19.00, 26.00		
Be aware of physical and emotional exhaustion	Yes	29.00, 38.00	−2.011	0.044	27.14 ± 5.625	0.386	0.719	19.00, 26.00	−1.387	0.166
	No	25.00, 35.00			27.39 ± 6.781			18.00, 24.00		
Be considerate of physical pain and sympathetic to sadness and grieving	Yes	29.00, 33.00	−2.907	0.004	27.09 ± 5.682	0.151	0.488	19.00, 22.00	−1.388	0.165
	No	25.00, 35.00			27.83 ± 6.119			18.00, 24.00		
Provide follow-up care after discharge from the hospital	Yes	30.00, 39.00	−2.699	0.007	26.78 ± 5.861	0.051	0.132	28.00, 37.00	−0.708	0.479
	No	28.00, 37.00			27.48 ± 5.592			19.00, 26.00		
Introduce to peer groups	Yes	28.75, 37.00	−1.172	0.241	28.73 ± 5.978	0.204	0.022	19.00, 28.00	−2.103	0.035
	No	28.75, 38.00			26.78 ± 5.603			18.00, 26.00		

Higher levels of BO were significantly associated with the following factors: being single (*p* ≤ 0.001) working in a general hospital (*p* ≤ 0.001), having no childbirth experience (*p* ≤ 0.001), having fewer than three childbirths (*p* = 0.013), no history of miscarriage (*p* = 0.048), absence of traumatic experiences (*p* = 0.004), receiving less than two types of organizational support (*p* = 0.004), a perinatal loss knowledge score below 5 (*p* = 0.005), lack of opportunities to create memories with the baby (*p* = 0.006), and not being referred to peer support groups (*p* = 0.022).

Higher STS scores were significantly associated with the following factors: being single (*p* = 0.021), having children (*p* = 0.023), working in general hospitals (*p* = 0.002), having no childbirth experience (*p* = 0.023), and not being referred to peer support groups (*p* = 0.035).

### Multiple linear stepwise regression

Several significant predictors of CS scores were identified, including possessing knowledge of GC for ≥5, working in OB/GYN, receiving support from the organization >2, and exhibiting higher resilience scores. These predictors accounted for 42.9% of the variance in CS scores (adjusted *R*^2^ = 0.429, *F* = 15.610, *p* < 0.001), with resilience being the strongest predictor (*p* < 0.001, *β* = 0.498) (Table 5).

**Table 5:** Multiple linear regression for ProQol.

Variables	Compassion satisfaction				
	*B*	Std. Error	*β*	*t*	*p*
(Constant)	4.065	2.406		1.689	0.092
Support from organization > 2	2.105	0.908	0.095	2.318	0.021
Knowledge of grief care > 5	2.096	0.952	0.131	2.202	0.029
OB/GYN hospitals/clinics	3.451	0.651	0.234	5.301	0.000
Resilience	0.294	0.025	0.498	11.883	0.000
Be considerate of physical pain and sympathetic to sadness and grieving	2.963	1.326	0.126	2.234	0.026
Adjusted *R*^2^ = 0.429, *F* = 15.610, *p* < 0.001					

Variables	Burnout				
	*B*	Std. Error	*β*	*t*	*p*
(Constant)	50.067	1.785		28.045	0.000
OB/GYN hospitals/clinics	−3.683	0.513	−0.300	−7.184	0.000
Resilience	−0.275	0.019	−0.560	−14.407	0.000
Single	1.614	0.811	0.122	1.989	0.048
Adjusted *R*^2^ = 0.491, *F* = 21.910, *p* < 0.001					

Variables	Secondary traumatic stress				
	*B*	Std. Error	*β*	*t*	*p*
(Constant)	35.453	2.213		16.018	0.000
OB/GYN hospitals/clinics	−1.393	0.617	−0.119	−2.258	0.025
Resilience	−0.173	0.023	−0.369	−7.443	0.000
Introduce to peer support group	1.472	0.722	0.107	2.039	0.042
University and above (Educational background)	−1.202	0.598	−0.105	−2.011	0.045
Adjusted *R*^2^ = 0. 169, *F* = 5.674, *p* < 0.001					

For BO, significant predictors included working in OB/GYN hospitals/clinics, resilience scores, and being single. These predictors explained 49.1% of the variance in BO (adjusted *R*^2^ = 0.491, *F* = 21.910, *p* < 0.001), with resilience being the strongest predictor (*p* < 0.001, *β* = −0.560).

Regarding STS, working in OB/GYN hospitals/clinics, resilience scores, introduce to peer support group and graduated from university and above accounted for 16.9% of the variance in STS (adjusted *R*^2^ = 0.169, *F* = 5.674, *p* < 0.001). Notably, resilience emerged as the strongest predictor across all three subscales (*p* < 0.001, *β* = −0.369).

## Discussion

Our survey obtained useful baseline data about the CF and CS of nursing staff caring for couples with pregnancy loss/infertility experiences, revealing unique risk and protective factors specific to this population.

Our study findings align with previous research, indicating moderate levels of compassion satisfaction (CS), burnout (BO), and low levels of secondary traumatic stress (STS) among nursing professionals (22, 23). In comparison to nurses working in ICU, oncology, emergency, and geriatrics departments, our participants exhibited lower CS and higher CF (24–26). This difference may be attributed to the impact of the Covid-19 pandemic, as some nurses and midwives were deployed to the front line, while the remaining nursing staff faced unprecedented pressure, potentially affecting their CF (22). Excessive patient load, staffing shortages, and a lack of training leading to low confidence were also identified as factors contributing to higher levels of CF in obstetric care (27). Furthermore, the unique context of maternity care—typically characterized by the joy of welcoming new life—creates a stark emotional contrast when managing pregnancy loss, potentially intensifying the sense of tragedy and emotional burden (27). This chronic exposure to trauma, shared by nurses in other high-mortality units, represents a significant challenge to professional well-being. However, compared to the termination of pregnancy providers working in South Africa and midwives working in rural districts of Uganda, our results showed higher CS and lower CF (8, 28). The possible reason is the background differences in economic, cultural, or staff team composition between different countries.

This study confirmed a negative relationship between CS and both BO and STS, consistent with previous research (22, 29). Our findings suggest that strategies aimed at enhancing CS may be associated with lower levels of CF. Additionally, resilience, another important construct in this study, exhibited a significant negative correlation with STS and BO, while a significant positive correlation was observed between CS and resilience, aligning with previous studies (30).

Consistent with some previous studies, this research showed that resilience exhibited the strongest negative correlation with CS and CF, and nurses with higher levels of resilience were less likely to experience CF (31). Resilience refers to the positive adaptation process under adversity, stressors, and traumatic events (32). Higher levels of resilience are associated with a more positive psychological state, indicating greater confidence in problem-solving and enhanced coping abilities to effectively manage and recover from work-related trauma, thereby dealing with CF more effectively and flexibly (31).

The sufficiency of GC knowledge positively associated with CS, consistent with the previous study (33). Nurses with sufficient GC knowledge provide confident and efficient care, leading to greater satisfaction (33).

The study found a negative association between working in a general hospital and CF, similar to a study in Japan where larger hospitals had higher BO and STS (34). Possible reasons: (1) Referral systems send complex cases to general hospitals, exposing nurses to pregnancy loss. Our research showed higher GC implementation rates in general hospitals (23.6% vs. 16.9 and 1.0%). Traumatic births bring emotional distress to nurses. (2) General hospitals lack teamwork, with only 30.2% having GC cooperation teams, the lowest among the three facilities. Cohesive teamwork improves care and may enhance CS (35). (3) High workload and nurse–patient ratio in general hospital led to BO and STS. Therefore, nurses working in general hospitals usually with a higher nurse–patient ratio, appear to experience higher levels of CF.

Organizational support was found to be strongly inversely associated with CF, consistent with prior studies (36). Support from organizations promotes midwives’ engagement in their work, thereby facilitating CS development.

Our study also found that single women were more susceptible to BO compared to their married counterparts, a result consistent with previous research. This association may be attributed to the relatively limited partner support available to single women (37, 38). Furthermore, higher educational levels were associated with lower levels of STS, which aligns with existing literature (39). One plausible explanation is that greater academic attainment is correlated with enhanced care-related knowledge and skills, thereby enabling more effective provision of assistance and potentially reducing emotional exhaustion.

The practice of GC is positively related to CF and negatively associated with STS. Although no existing study directly addresses this aspect, a similar investigation on pediatric palliative care has consistently reported comparable findings (40). Our study found that expressing compassion for patients’ physical and psychological suffering can enhance CS, consist to the result of a previous study (23). This may be attributed to the inherent rewards of the nursing role: by fulfilling their core responsibility of providing help and care, and witnessing patients receive comfort, nurses often develop a profound sense of professional efficacy and accomplishment, thereby strengthening the positive psychological rewards derived from caregiving. The altruistic nature of nursing in Japan is reflected in the willingness of nurses (66.9%) to provide GC to help others (41). The pleasure derived from helping others, reflected in higher levels of CS. Thus, the meaningful and worthwhile nature of GC, related to nursing culture, helps prevent exhaustion among healthcare professionals.

Our research revealed that nurses might experience STS when introducing patients to peer support organizations. This could be attributed to nurses’ limited information and knowledge in this area. Survey results indicated that only 57.6% of nurses and midwives were aware of organizations offering peer support, which was the lowest among the assessed items (42).

In order to reduce burnout and improve the quality of care for pregnancy loss/infertility, clinical care managers should consider the following aspects. Firstly, prioritizing organizational support and implementing well-designed working schedules is essential. Secondly, it is crucial to pay attention to the resilience of nurses from general hospitals and provide relevant support. Thirdly, establishing standardized protocols for emotional care related to pregnancy loss/infertility is significant. Lastly, hospitals and educational institutions should collaborate to strengthen training programs for nursing students, emphasizing the development of emotional care skills in this specific area. These measures will contribute to reducing CF and improving the overall quality of care for patients experiencing pregnancy loss/infertility.

### Strengths and limitations

Our study has several limitations. The convenience sampling approach might have led to potentially biased estimates. The use of only quantitative data means that causal relationships cannot be inferred. Lastly, some measures tended to be subjective as the surveys were self-completed. Future research should test these findings on a large scale across multiple centers. Qualitative studies are also desirable, to broaden our understanding of bereavement care and design possible interventions. Identifying the prevalence and possible predictors of CF and CS among nurses/midwives in other parts of the world would be significant.

## Conclusion

This study identified key factors influencing CF and CS among nurses caring for patients with pregnancy loss and infertility. Findings indicate that psychological resilience, knowledge and organizational support are central modifiable elements. We recommend that healthcare institutions implement targeted resilience training programs, establish structured peer-support systems, and integrate compassion practice training into continuing education. Nursing managers should acknowledge the distinctive emotional demands of this specialty and foster supportive workplace environments to enhance professional quality of life. Future research should focus on developing and evaluating tailored interventions for this vulnerable nursing population.

## Data Availability

The raw data supporting the conclusions of this article will be made available by the authors, without undue reservation.
